# A Review on Newer Ocular Drug Delivery Systems with an Emphasis on Glaucoma

**DOI:** 10.34172/apb.2021.048

**Published:** 2020-09-19

**Authors:** Maneesha Peter, Rajitha Panonnummal

**Affiliations:** Amrita School of Pharmacy, Amrita Institute of Medical Science & Research Centre, Amrita Vishwa Vidyapeetham, Kochi-682041, India.

**Keywords:** Glaucoma, Intraocular pressure, Aqueous humour, Nanoformulations

## Abstract

Glaucoma is an irreversible condition resulting from the increase in intraocular pressure (IOP); which leads to permanent loss of vision with the destruction of retinal ganglion cells (RGCs). The IOP elevations are controlled in normal by the physiological flow of aqueous humour. A population with age above 40 is more susceptible to glaucoma. Other factors like gender, genetics, race etc. plays major roles in the development of the disease. Current treatment methods available for the disease includes drugs come under the classes of beta receptor blockers, carbonic anhydrase inhibitors, cholinergic agonists, prostaglandins etc. N-methyl-D-aspartate (NMDA) antagonists, inducible nitric oxide synthase (iNOS) inhibition, cytoskeletal agents, Rho-kinase inhibitors etc are few novel targets sites which are in research focus for the treatment of the disease. Developments in nanomedicine are also being evaluated for their potential in treating the growing glaucomatous population. Nanosystems are suggested to avoid the difficulties in tackling the various ocular barriers to a limit, help to decrease the instillation frequency of topical medication and can provide drug delivery in a sustained or controlled manner. This review focuses on the current and emerging treatment methods for glaucoma along with some of the nanoformulations for ocular drug delivery.

## Introduction

**Figure 1:**
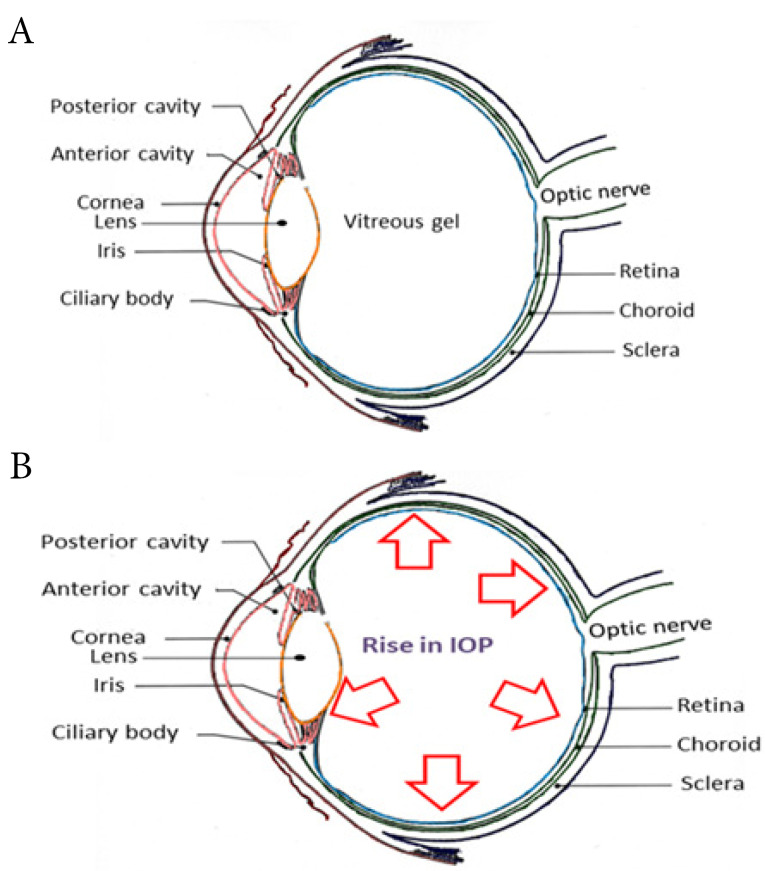
Schematic representation of (A) Healthy eye & (B) Eye affected with glaucoma

Eye forms an integral component of the sensory system with highly advanced anatomy and physiology. The different segments of the eye work in harmony to facilitate vision. Human eye is distinctively categorized into an anterior chamber, posterior chamber and the vitreous cavity as depicted in Figure 1A. The outer layers of the eye consist of the sclera and cornea. The former maintains the structural configuration and attaches eye to the extrinsic muscles. The cornea is noted as a clear membrane that extends along with the sclera and manifests as the outer fibrous layers of the eye. The vascular layers are formed by the choroid, ciliary body and iris.^1,2^ Choroid operates by absorbing the light rays which fall onto the retina. Ciliary bodies are innervated with a system of parasympathetic nerves and it also renders the attachment to the suspensory ligaments. Contractions and relaxations of the ciliary muscles assist in the bending and entry of the light rays, so that it can be further focused onto the retina. Aqueous humour, which is released into the anterior segment of the eye, is produced by the ciliary body. The watery fluid generated from the ciliary body is filtered by the posterior chambers and drained out through the cells of the trabecular meshwork.^3,4^ Lying behind the cornea and in front of the lens is the iris, which divides the eye into anterior and posterior chambers. Iris is composed of circular and radial muscle along with an aperture in the middle, named as pupil.^5^ Pupil can change its size depending on the intensity of the light entering into the eye. Behind the pupil lies the lens, which is biconvex and is orchestrated by the suspensory ligaments.^6,7^ It is followed by the innermost layer, retina. It is constituted by a multitude of neurons. Retina accommodates rods and cones which forms the light-sensitive part. Blood supply to the eye is enabled by the ciliary arteries and the central arteries of retina. The jelly-like, vitreous body is formed beyond the lens, helps to maintain the structure of the eye and avert the walls of eyeball from collapsing.^8,9^ Along with these central regions that control sight, eye is equipped with certain accessory structures like the eyebrows, eyelids and eyelashes that protect the eye from injuries and high luminescence. Along with them, the conjunctiva lines the eyelids and acts as a protectant of the cornea. Lacrimal glands secrete tears composed of salts, water and lysozyme which wipes and nourishes the front of the eye.^3,10^ The optic nerve emerges from the retina, converges to the side of macula lutea, passes through the optic foramen and meets at the optic chiasma. As the light rays entered into the eye, the pupil size is varied. The entry of bright light constricts the pupils and the pupils dilate when less intense light is detected. Pupils restrict the entry of high amounts of light and thereby prevent the damage to the retina. Also, the eyeball moves inside (convergence) so that the nearer objects are easily visible.^3^ In order to view a distant object, the convergence produced by the eyeball is less. Brighter lights are required to impart the stimulation of cones and thereby the perception of vision. The lights rays which entered into the eye introduce reactions which are converted into nerve impulses and transmitted through the optic nerve, to aid vision.^4^

Glaucoma is an irrevocable condition that emerges as a result of the increase in intraocular pressure (IOP) (more than 20 mm Hg) from the normal pressure of 10-20 mm Hg (Figure 1B) along with the regression of retinal ganglion cell (RGC) layers.^11^ The IOP elevations in glaucoma occur as a result of increased resistance to aqueous humour outflow and are recognized as one of the paramount inducers of detrimental effect on vision.^12,13^ Glaucoma is divided into primary, secondary, congenital, pigmentary and normal-tension glaucoma (Table 1).^14^

**Table 1:** Different categories of glaucoma

**Type**	**Definition**
Primary glaucoma	Arises due to an unknown cause
Secondary glaucoma	Due to any defined cause for the increase in intraocular pressure, e.g. eye injury, inflammation etc.
Congenital glaucoma	A congenital defect which may be inherited
Pigmentary glaucoma	Pigments of the iris flake off and clog the drain outlet of the aqueous humour
Normal-tension glaucoma	Damage to the optic nerve even when the pressure is normal

Glaucoma is again subdivided based on the iridocorneal angle into:

i) Open-angle glaucoma/Wide-angle glaucoma 

ii) Closed-angle glaucoma/Narrow-angle glaucoma 

When the iridocorneal angle is intact but the flow of the aqueous humour through the trabecular meshwork is disturbed due to any degeneration or disturbance in the meshwork system, it is termed as open-angle glaucoma. The condition is asymptomatic and may be chronic. Closed-angle glaucoma is defined as a state when the iridocorneal angle is closed and the flow of aqueous humour from posterior to anterior chamber is disrupted. The condition is manifested with pain and pressure build-up in the eye.^15,16^

## Pathophysiology

**Figure 2:**
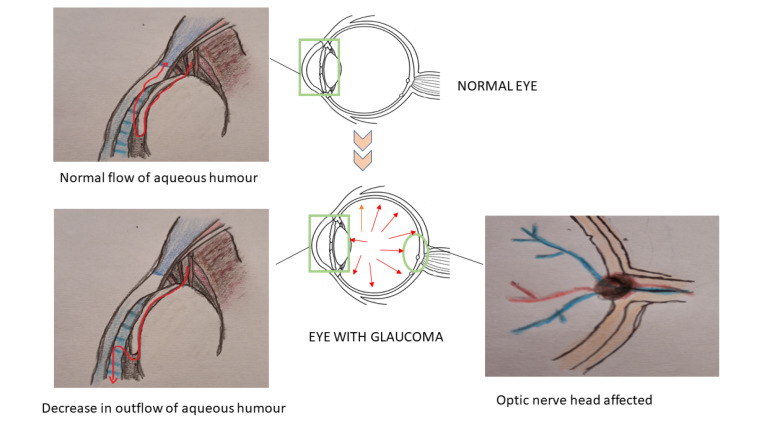
Schematic representation of glaucoma and its main defects

Normally the aqueous humour produced by the ciliary body is drained out through the trabecular meshwork and the Schlemm’s canal. Some amount of the aqueous fluid also passes through the uveoscleral pathway; this decreases the build-up of pressure inside the eye. But in glaucomatous condition, the systematic outflow of the aqueous humour is altered due to any insult in the trabecular meshwork or any changes in the iridocorneal angle. This may result in the obstruction of the aqueous humour outflow with pressure build-up and damages the optic

nerve head (Figure 2). Studies also indicate that the presence of mutant form of myocilin (myocilin is a protein secreted into the aqueous humour and its function is not clear) deposits in the trabecular system initiates a cascade of toxic events that may result in higher IOP.^16,17^

Damage to the RGCs is reported to be involved in the development of glaucoma. The altered appearance of the optic disc also speaks for the same. An escalation in the IOP, causes the fluid to move from the vitreous cavity to the extracellular spaces, thereby resulting in rapid necrosis of the neuronal bunches. Deprivation of the visual field is due to the trauma to the optic nerve head (ONH). The elevated IOP also damages the RGCs as revealed by the depletion of the rate of the axonal transport. The glial cells in the ONH are activated due to high IOP and this leads to the remodelling of the extracellular matrix (ECM) and its characteristic degradation. These alterations result in the amplification of tension on the RGC axon.^18,19^

An imbalance between the generation and expulsion of the free radicals result in a state denominated as oxidative stress.^20,21^ It affects the vascular flux of the optic nerves, produces injury to the trabecular meshwork and initiate an aberrant immune response with glial cells dysfunction. Activation of the apoptotic pathway by reactive oxygen species further contribute to the death of RGC.^22^ Decreased amount of oxygen in the retina stimulates hypoxia-inducible factor 1ɑ which in turn exerts protective effects on RGC. Free radicals also activate biological pathways of NF-κB (nuclear factor kappa-light-chain-enhancer of activated B cells), IL-1 (interleukin 1), ELAM- 1 (endothelial cell leukocyte adhesion molecule-1), TNF-ɑ (tumor necrosis factor-α) etc contribute to neuronal death.^23,24^

## Risk factors

Distinguishing glaucomatous population is a tough endeavour as the illness is identified only after the diminution of a notable percent of RGCs. Evidences revealed that a senile community, Africans, Americans, familial history of glaucoma, raised IOP, reduced diastolic perfusion pressure and myopia as cardinal risk factors for glaucoma.

## Age

Age-related susceptibility can be perceived as a supreme influencer in the rise of the glaucomatous population. Patients aged 40 and above are more prone to developing glaucoma.^25,26^

## Gender

As per reports, the female population is more prone to get affected by glaucoma, particularly angle-closure glaucoma.^27^

## Familial history

Research works revealed that the persons having a first degree relative with glaucoma there is four times risk for getting the disease when compared with persons without such a relative.^26^

## Genetics

GLC1A is a gene coding for a molecule that is available in the aqueous outflow channels. Mutation in these passages fosters an occlusion in the outflow leading to a higher IOP. Both the trabecular meshwork and ciliary bodies monitor the IOP and contain myocilin fraction. MYOC locus in the GLC1A codes for myocilin and mutations in this locus is considered as a contributor factor for 3%-5% of adult-onset primary open-angle glaucoma.^26^

## Ethnicity

Glaucoma is being considered as one of the significant causes of blindness among African Americans, Caucasians etc.^26^

## Treatment

Drug therapy, laser and surgical methods are also utilized to reduce IOP. The judgment of the correct therapy for the treatment of the disease is influenced by certain criteria like IOP levels, stage and advancement of diseases, treatment methodologies followed etc. Along with all these factors, satisfaction with the therapy provided and patient compliance forms the basis for choosing the right treatment. The conventional therapy employed mainly relies on topical administration of the drugs as eye drops or ointments.

## Beta-blockers

β blockers act by competitively inhibiting adrenergic β receptors and thereby decreasing the secretion of aqueous humour. Topical β blockers are either non-selective or β_1_selective in performance. Non-selective β blockers suppress the action of adrenergic agonist on both β_1_ and β_2_ receptors.^28,29^

## Topical carbonic anhydrase inhibitors

Dorzolamide, Brinzolamide etc. lower the IOP by minimizing the aqueous humour production by hindering the sodium pump and is often used as adjuvant in therapy. The adverse events reported with the instillation of topical carbonic anhydrase inhibitors are burning and stinging sensation, bad taste in the mouth and conjunctivitis.^30-32^

## Cholinergic agonists

Parasympathomimetic agents like pilocarpine are rarely used as first-line therapy. They act on the iris, reduce contraction of the ciliary muscle and improve the IOP.^31,32^

## ɑ_2_ agonists

Brimonidine is a highly selective and potent ɑ_2_ adrenergic receptor agonist that lowers IOP by promoting uveoscleral outflow of aqueous humour and by decreasing its production. Studies showed that brimonidine 0.2% can significantly decrease IOP in patients with uncontrolled primary open-angle glaucoma when used as an adjuvant along with dorzolamide and is also having neuroprotective effect.^33,34^

## Prostaglandins

Prostaglandins act by relaxing the muscles in the inner segments of the eye, thereby enabling efficient outflow of aqueous humour and thus prevent the escalation of ocular pressure. Possible side effects associated with the use of prostaglandins include stinging and burning, eye colour change etc.^33-35^

## Rho-kinase inhibitors

A newly developed agent in the topical treatment for glaucoma is the Rho-kinase inhibitors. The enzyme, Rho kinase regulates the calcium-independent contractions of smooth muscles. Reports stated that these enzymes can affect the cytoskeletal aspects like morphology, cell adhesion etc. Rho-kinase inhibitors can reduce the IOP by increasing the flow of aqueous humour through the modification of the structure of the trabecular meshwork system.^31-35^

Due to the asymptomatic and chronic nature of the disease, patient compliance is necessary to prevent the progress of the disease and to ensure medication adherence for long periods.^30^ Treatment often involves multiple drug therapies with the potential for adverse interactions including the development of ocular surface disorders. The justification for non-adherence to the treatment partly lies in the discomfort of eyes by the use of these medications. Laser treatment and trabeculectomy are considered as alternatives to drug therapy.^36^ Argon laser trabeculoplasty & micro pulse laser trabeculoplasty are few examples of this. Other relatively newer techniques employed are viscocanalostomy and high frequency deep sclerectomy.^37,38^

## Barriers to treatment

### Tear

To counteract the irritations produced on the eye, lacrimal glands secrete lacrimal fluid or tears and the mean volume of production is about 7 to 9 mL. On installation of eye drops into the eye, the instantaneous increase in tear volume causes excessive medication to spill out.^39,40^

### Cornea

The corneal epithelium acts as a powerful barrier for the drug to pass. The epithelium is a highly lipophilic tissue and makes is difficult for the hydrophilic drug moieties to cross this barrier.^39-41^

### Conjunctiva

The conjunctiva is composed of 5-15 sheets of stratified squamous epithelium cell layers. It is supplied with lot of the nerves, lymphatic and blood vessels. The permeability of drug to the conjunctiva is higher when compared with that of sclera and cornea.^42-44^

### Blood retinal barrier (BRB)

BRB functions as a barrier between the blood and the retina and contains tight junctions. As a result of the anatomical position of the BRB, it effectively limits the transportation of molecules from the choroidal blood circulation to the posterior segment of the eye.^40,43^ On topical application, the primary aspect to be noted is that the drug needs to be penetrated. Lipophilic moieties and unionized fractions are better penetrated compared to a hydrophilic agent or an ionized drug. The drug needs to be delivered to the target site using a vehicle & the vehicle used influences the retention time of the agent in the conjunctival cul-de-sac.^45^ Elevation of retention time increases the trans corneal diffusion and lowers the systematic level of the drug. The viscosity of ophthalmic products can be made adequate by the inclusion of compounds such as hydroxyl propyl methylcellulose, polyvinyl alcohol etc. These polymers increase the residence time of the drug in the conjunctival cul-de-sac and offers a slower clearance resulting in enhanced absorption of the drug moiety.^46^

## Novel therapeutic targets in glaucoma

Even in this era of modern medicine, glaucoma still remains as a major cause of blindness worldwide. Therefore, newer therapeutic strategies targeting the alternative pathways involved in disease genesis and progression need to be identified.

## NMDA antagonists

Stimulation of the glutamate N-methyl-D-aspartate (NMDA) receptor triggers the opening of ion channels and permits the entry of extracellular calcium and sodium. Extracellular calcium acts as a secondary messenger to initiate neuronal cell death. To suppress excitotoxicity mediated cell death, efficient removal of synaptic glutamate is essential.^47^ The synapses are encircled by Muller cells and astrocytes which transport glutamate (extracellular) into the glial cells. Glutamine synthetase converts glutamate to glutamine (non-toxic) which is taken up by the neuronal cells and is again converted to glutamate in the presence of glutaminase thereby preventing the glutamate toxicity by maintaining the physiological neurotransmitter stores.^48,49^

## Cytoskeletal agents

Alterations to the trabecular meshwork cytoskeleton can change the local geometric outflow pathway and consequently alter the aqueous outflow. Compounds having the property to causes cytoskeletal alterations are found to effectively lower IOP. For example, cytochalasin, prevent the elongation of actin filaments, causing distension of the trabecular meshwork, rupture the inner walls of the Schlemm’s canal and increase the aqueous humour outflow.^47,48^

## Matrix metalloproteinase inducers

These agents are capable to catalyse the hydrolysis of ECM proteins; as higher concentration of ECM in the trabecular meshwork causes resistance for the aqueous humour outflow.^47,49^

## iNOS inhibition

Nitric oxide synthases (NOSs) are enzymes that catalyse the synthesis of nitric oxides. An elevated level of iNOS (inducible-NOS 2) is observed in the glaucomatous population. These findings point out the accumulation of toxic amounts of NO, leading to the development of peroxy radicals; which is capable to induce inflammations and neuronal damage. Therefore inhibition of iNOS is an important strategy to reduce inflammation and neural damage; hence it serves as a potential tool in the treatment of glaucoma.^31,49^

## Neuroprotectives in glaucoma

The complexity of mechanisms which transform to the formation of neuronal damage in glaucoma has to be dealt with utmost perceptiveness. Neurons in the visual pathway are deprived due to the progressive damage caused by the rise in ocular tension.^50^ RGCs are the primary victims affected by this process. Genetic factors, withdrawal of neurotropic factors, exposure to reactive oxygen species etc. are the main causative agents expected to be involved in RGC degeneration.^18,51,52^ Some of the compounds having neuroprotective property and thus improves RGCs survival are mentioned in Table 2.^53^

**Table 2:** Different compounds which provide neuroprotection in glaucoma

**Compound**	**Mode of action**
CoQ10	Effect on mitochondrial dysfunction
Ginkgo biloba	Antioxidant
Melatonin	Antioxidant
Minocycline	Anti-inflammatory
Resveratrol	Antioxidant
Citicoline	Neuroprotective
Brimonidine tartrate	Neuroprotective

## Brimonidine

Brimonidine acts as a successful α_2_ adrenergic agonist which is in use for the treatment of glaucoma. Research works illustrate that 0.2% brimonidine tartrate solution is capable to activate the α_2_ receptors present in the retina.^54^ WoldeMussieet al studied the neuroprotective effect of brimonidine on RGCs in rats treated with laser- induced chronic ocular model of hypertension. The investigation is able to conclude that the brimonidine is able to decrease ganglion cell death by 26±1% and 15±2% at doses of 0.5 and 1 mg/kg respectively.^55^ Ferencz et al studied the neuroprotective effect of topically applied brimonidine in patients undergone laser treatment for choroidal neovascularization and reported the significant improvement in vision.^56^

## Citicoline

Citicoline or cytidine 5’-diphosphocholine is an endogenous compound made up of ribose, pyrophosphate, cytosine and choline. It is a nontoxic moiety which increases the brain neurotransmitters such as dopamine, noradrenaline, serotonin etc.^57^ It also acts as an intermediate in the synthesis of CNS phospholipids and also the drug is believed to improve the synthesis of phosphatidylcholine.^58^ Oshitari et al reported the neuroprotective effect of citicoline in 2002. TUNEL staining (terminal deoxynucleotidyl transferase dUTP nick end labelling) is done to explore the consequence of the administration of citicoline to mouse retinal explants. An obvious expansion in the number of neurons or regeneration is observed after the administration of citicoline.^59^ Ottobelli et alevaluated the consequence of oral use of citicoline on visual field rates in 41 patients with progressing glaucoma and reported the decreased progression of the disease in these patients, indicates its neuroprotective effects.^60^

## Gingko biloba

Gingko biloba extracts is typically composed of flavonoids and terpenoids. EGb761 and LI1370 are reported to be isolated from the extracts possessing antioxidant properties.^61^ Moreover, the extracts are reported to cause vasodilation, thereby improve the blood flow and safeguard mitochondrial metabolic process.^62^ Quaranta et al performed a double-masked randomized crossover trial with 27 patients to identify the effect of Gingko biloba extract on pre-existing visual field damage in normal-tension glaucoma and reported the improved visual field damage.^63^

## Ubiquinone/Coenzyme Q 10 (CoQ10)

Ubiquinone acts as a cofactor in the mitochondrial electron transport chain, which is responsible for the transport of electrons from complex I &II to complex III.^64,65^ Mitochondrial dysfunction is reported to be involved in apoptosis of neurons and ubiquinone can counteract these effects.^53^ A study was conducted by Lee et al to investigate the neuroprotective effect of ubiquinone on RGCs in glutamate excitotoxicity and oxidative stress model in DBA/2J mice. CoQ10 is found to boost the growth of neurons in the ONH by 29%, as well as decreases the apoptotic cell death by suppressing the expression of proapoptotic Bax (Bcl-2-associated X protein) or by enhancing the expression of antiapoptotic Bad protein.^66^ Another study conducted by M. Cordeiro *et al* reported the decreased RGC death on topical application of CoQ10 in an experimental model of glaucoma, suggested it as a promising candidate for use in glaucoma.^67^

## Resveratrol

Resveratrol is a plant polyphenol commonly found in grapes. Chemically it is a stilbenoid and is considered as a phytoalexin generated by various plants in response to injury or when attacked by pathogens.^68^ Luna et al carried out a study to reveal its effect on the expression of glaucoma markers induced by chronic oxidative stress and is reported that resveratrol limited the expression of inflammatory markers like IL-1α, IL-6, IL-8, etc., along with a decrease in senescence marker lipofuscin manifested in glaucomatous condition. Resveratrol is effective in repairing the abnormalities associated with trabecular meshwork cells in primary open-angle glaucoma.^69^ It is also reported that the resveratrol can induce a delay in RGC death in hyaluronic acid (HA) model of glaucoma.^70^

## Novel ocular drug delivery approaches

Glaucoma is said to affect around 111.8 million people by the year 2040.^71^ Therefore, the requirement of a novel therapeutic strategy is the need of the hour. A variety of factors are there to influence the manufacturing of drug delivery systems and novel delivery approaches. These factors need to be considered much to develop ideal delivery systems for the treatment of glaucoma.

## Mucoadhesive polymers

Use of the right polymer or a mixture of polymers is essential to improve the ocular residence period as well as the bioavailability.^72^ Polymers used in the preparation of formulations play a key role in stabilizing the carrier systems.^73^ These polymers are categorized into biodegradable and non- biodegradable polymers. Biodegradable polymers have a short duration of action compared to non-biodegradable forms.^74,75^ These polymers should possess good viscosity, adherence and a reduction in the drug drainage rate. Attachment of drug carrier with the mucin coat covering the conjunctiva & cornea elevates the drug residence time, establishes a contact between the drug and the tissue, which absorbs it. This results in a higher drug concentration at the target.^76^ Hyaluronic acid (HA) is a natural glycosaminoglycan remains as the first among these polymers. This mucoadhesive biological polymer has the advantage of having a highwater binding capacity, low irritancy, better viscosity and pseudoplastic behaviour.^77,78^ Other mucoadhesive polymers include carboxymethylcellulose, polyacrylic acid derivatives, xanthan gum, carrageenan etc.^78,79^ Chitosan is a polysaccharide obtained from chitin by deacetylation and has several features that make it favourable for the construction of a controlled drug delivery devices to the eye.^80,81^ It is a hydrophilic mucoadhesive polymer which is biocompatible and biodegradable with good eye tolerability. Moreover, chitosan exhibits antimicrobial and wound healing properties.^82^ It is reported that the positively charged amino groups of chitosan associates with the negatively charged sialic acid residues of mucin, thereby prolonging the contact time of the drug. It is a widely utilized polymer for application in various drug targeting methodologies and also acts as an excipient in vaccine delivery, gene therapy etc. Chitosan offers sufficient muco-adhesiveness, ocular permeability, biocompatibility and corneal wound healing along with antibacterial and antifungal effects.^83^ It can easily turn to gels by physico-chemical methods or through co-ordination linkages without the need of any additives. Chitosan in situ gelling systems are used to deliver bioactive compounds by instillation into the eye, which upon exposure to the ocular media changes to gel.^84^ These are liquid at room temperature and transform into gel after application to the ocular surface due to changes in temperature, pH or ionic strength. Chitosan may interact with water-soluble macromolecules such as anionic polysaccharides, dextran sulphate, collagen or anionic polymers such as polyacrylic acid.^85^

Carbopol is a polyacrylic acid derivative which initiates sol-gel transition in aqueous solution when the pH of the medium rises above 5.5. It is non-toxic and non-irritating to humans following topical application. However, the concentration required to form gel resulted in acidic solutions that cannot be rapidly neutralized by lacrimal fluid buffers.^86^ Chitosan can be complexed with Carbopol through the electrostatic interactions between the amino groups of chitosan and the carboxyl groups of carbopol. Cross-linking of chitosan is imperative to ameliorate its properties like stability and durability for drug delivery.^87^

Various other polymers like collagen, pluronic F-127, polyvinyl alcohol etc are utilized widely in manufacturing novel drug delivery systems. Collagen is a naturally occurring polymer reported to have optimum strength and low toxicity.^88^ Pluronic F127 is also called as Poloxamer 407; contains both polar and non-polar substituent in it and is amphiphilic in nature. It possesses good gelation property but it is difficult to maintain its stability.^89^

## Contact lenses

One of the major problems associated with glaucoma is low medication adherence. The topical medications need to be instilled two or three times a day. This issue can also be managed by the usage of contact lenses as they can provide a controlled or sustained release of medication.^90^ Thus the problems of low ocular availability and less residence time of the drug carrier system can be tackled by using medicated contact lenses. Both conventional and silicone hydrogel contact lenses release ophthalmic drugs in a short period.^91^ But nanoparticle laden lenses, biomimetic and imprinted contact lenses with layered structures may permit an extended drug delivery along with improved ocular availability and reduced side effects. The usage of contact lenses in glaucomatous patients is reported to achieve the same efficacy which is comparable with other therapeutical regimens.^92,93^ Peng et al state that contact lenses with only 20% of the drug loading are able to achieve the same efficacy as that achieved with eye drops. A significant lowering of IOP is achieved by the continuous usage of it.^94^ Hsu et al formulated contact lenses loaded with both dorzolamide and timolol and the system is reported to prolong the release of the loaded moieties for 2 days with sufficient decrease in IOP when compared to eye drops. Also, the system in combination with Vitamin E is proved to be more beneficial due to the antioxidant potential of vitamin E.^95^

## Ophthalmic inserts

These are solid gadgets available to place into the conjunctiva of eye designed to induce the release of drug for an extended time frame at a constant rate to improve patient compliance.^96,97^ Ophthalmic inserts also promote non-corneal diffusion. But the systems are reported to cause discomfort among its users as these are not dissolving in the conjunctival sac.^98^ This factor encourages the development of non-irritant, non-toxic, more convenient and soluble the ophthalmic inserts by using polymers like polyacrylamide, polyvinyl pyrrolidineetc.^99^

## Iontophoresis

Ocular drug delivery can be achieved through non-invasive techniques like iontophoresis. In this technique a weak current supply the charged species through the ocular tissue and another ground electrode is placed elsewhere on the body to complete the circuit.^100^ Medications are retained in the iontophoretic device by two methods, either as a solution or as saturated in a gel. Ocular iontophoresis is achieved by trans corneal or transscleral iontophoresis. Trans corneally the drug is delivered onto the anterior segment and is suggested to be beneficial in diseases like glaucoma, inflammations, keratitis etc.^101,102^ Transscleral drug delivery helps in the administration of high concentrations of the drug into the inner or posterior segments of the eye. Current density and iontophoretic exposure intervals influence the drug delivery to the various regions of the eye. The procedure emphasizes minimal discomfort for the patient; however, it possesses several disadvantages such as epithelial oedema, burns, damage to the site of application etc.^103^ The technique is also limited to drugs having a small size; ionic nature and lower molecular weight.^104^

## Nanoformulations

**Figure 3:**
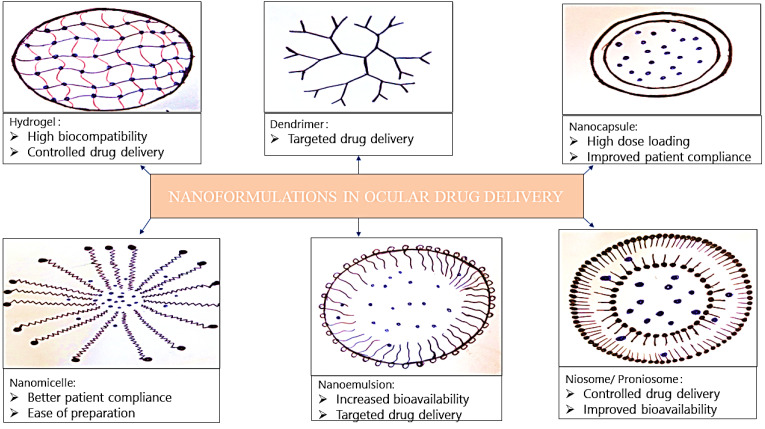
Nanoformulations used in ocular drug delivery.

Recently different nanoformulations are reported to have favourable properties for ideal use for ophthalmic applications. The size of different nanoformulations is usually below 1000 nm. These are fabricated through chemical processes to control the release of therapeutic agents and to enhance the ocular penetration. The size of the complex drug particles in an ophthalmic preparation should be less than 10 µm to avoid ocular irritation. The major advantages of utilizing nanocarriers in the treatment of ocular diseases are to enhance the bioavailability of topical administration, to achieve a controlled release, permit targeted drug delivery and ultimately to achieve improved therapeutic efficacy.^105,106^ Nanocarriers also offers higher drug retention time, lower dosage requirements, decreased dosing frequency and higher patient compliance (Figure 3). Cornea and conjunctiva possess negative surface charges and enhances the retention time of positively charged nanoparticles more efficiently than the anionic carriers, providing an increased opportunity for the drug to enter into the eye.^107,108^ Nanoparticles are becoming a resourceful therapeutic agent in eradicating various disorders associated with the posterior and anterior regions of the eye (Table 3).^109^

**Table 3:** Nanoformulations reported for ocular drug delivery

**Formulation**	**Drug loaded**	**Polymers used**	**Method of preparation**
Nano micelle	a) Methazolamide b) Rapamycin c) Cyclosporin A	a) Methoxy-poly (ethylene glycol)-b-poly(ε-caprolactone) b) Vitamin E tocopherol polyethylene glycol succinate&octoxynol-40 c)Polyvinyl caprolactam-polyvinyl acetate-polyethylene glycol & Pluronic F127	a) Thin film hydration b) Solvent evaporation c)Solvent evaporation/ Film hydration
Nano emulsion	a) Dorzolamide b) Moxifloxacin c) Acetazolamide	a) Poloxamer 407 b) Soluphor® P c) Peanut oil	
Nano capsule	a) Pilocarpine b) Prednisolone c) Pilocarpine	a) Poly(ε-caprolactone) & Pluronic F68 b) Poly(ε-caprolactone) or Eudragit RS100 c) Poly isobutyl cyanoacrylate	a) Double emulsion b) Interfacial deposition c) Interfacial polymerization
Dendrimer	a) Brimonidine & Timolol b) Fluocinolone c) Timolol analogue	a) Polyethylene glycol b) Hydroxyl-terminated poly amido amine c)Heterobifunctional amine polyethylene glycol acetic acid	
Hydrogel	a) Timolol b) Cannabigerolic acid c) Bevacizumab	a) Propoxylated glyceryl triacrylate b) Hyaluronic acid & methyl cellulose c) Glycol chitosan & oxidized alginate	a) Thermal polymerization
Proniosome/Niosome	a) Lomefloxacin b) brimonidine c) Dorzolamide	a) Cholesterol b) L-α-phosphatidylcholine & Cholesterol c) L-α-lecithin & Cholesterol	a) Coacervation phase separation b) Coacervation phase separation c) Coacervation phase separation

## Nanomicelles

Agents that are amphiphilic and capable of self-assembling above the critical micellar concentrations are termed as nanomicelles; measuring about 10–100 nm in size.^110,111^ Nanomicelles consists of a hydrophobic core where the drug is confined and the hydrophilic tail protrudes to the outside environment.^112^ Nanomicelles are advantageous as they maintain an ideal concentration of the medicament in the respected targets, better patient compliance, easy to prepare, small in size and improves the availability of medications in visual tissues.^113^ The utility of micellar scale preparations are limited as it may experience rapid tear dilution, untimely drug release & cost.^114^ Elmowafy et al reported the possibility of a better evolved therapy in glaucoma using nanomicelles. The authors converted methazolamide, a carbonic anhydrase inhibitor into a polymeric micelle which is found to improve the drug entrapment efficiency and ocular tolerability. The formulation showed an initial burst followed by a sustained release.^115^ Rapamycin loaded nanomicellar formulation for the delivery to the posterior segment of the eye is reported by Cholkar et al. The formulation is reported to have a size of approximately 10 nm and could transport the rapamycin into the retina and choroid following topical application. The *in vitro*cytotoxicity analysis demonstrated very little cytotoxic effects.^116^ Guo et al fabricated cyclosporine A loaded nanomicellar formulation using PVCL-PVA-PEG copolymer to achieve efficient drug delivery to treat immune-mediated corneal disease. This nanoformulation is found to possess good tolerance, stability on storage, no cytotoxicity and adequate ocular tolerance. Multiple and even single instillations of the formulation delivered promising levels of cyclosporine A into theeye.^117^

## Nanoemulsions

Nanoemulsions are thermodynamically stable drug carriers within the size range of 10-1000 nm with an interfacial surfactant layer separating the oil and water environments.^118^ These drug carriers are identified as oil in water, water in oil and bicontinuous nanoemulsions. Emulsions and nanoemulsions are easily identified from their appearance as the former tend to be cloudy in nature but the latter is translucent.^119^ Compared to other possible drug carrier systems, nanoemulsions offer increased drug bioavailability, improved drug absorption, rapid drug penetration with the protection of medicament from chemical reactions like hydrolysis and oxidation.^120^ Nanoemulsions allow the entrapment of hydrophilic and lipophilic drug particles. Besides, it is easy to adjust the dose of the drugs without altering the efficacy of the system.^121^ Manufacturing of nanoemulsions can often be allotted as an intricate process as precise temperature and pH conditions have to be maintained to ensure the stability of the preparation. Along with that, the excipients used especially the surfactant concentrations need to be adjusted so as to be non-toxic to the human tissues.^122^ Dorzolamide hydrochloride loaded in situ gel nanoemulsion is developed by Ammar et al. Nanoemulsion form of the drug is found to have longer ocular residence time and therapeutic potential when compared with the free drug solution. This formulation offers a lesser frequency of instillation of dorzolamide and intensified the action of the medication.^123^ Shah et al formulated nanoemulsion loaded with moxifloxacin*. In vivo* studies are conducted on rabbit models to evaluate the pharmacokinetic properties. The formulation showed good tolerance to toxicity, improved antibacterial efficacy and prolonged ocular availability. The emulsion system exhibited appropriate viscosity and improved stability.^124^ Nanoemulsion based electrolyte triggered in situ gel for ocular delivery of acetazolamide is reported by Morsi et al. In the work acetazolamide loaded nanoemulsions is prepared using peanut oil, tween 8- and or cremophor EL along with transcutol P or propylene glycol as co-surfactant. This is incorporated into an in-situ gelling system using gellan/xanthan. Formulation exhibited better stability and improved IOP lowering effect when compared with the marketed formulation of brinzolamide and oral acetazolamide tablets.^125^

## Nanocapsules

Nanocapsules are a drug delivery system in which the drug is enclosed inside a polymeric membrane or protective matrix with size starting from 10 nm. The drug is entrapped, adsorbed or dissolved into the matrix system.^126^ It is one of the efficient means of drug delivery system and is considered more advantageous than the free drug counterparts with improved bioavailability, sustained release, decreased toxicity and greater reproducibility. Nanocapsules markedly reduce the drug content required to acquire maximum therapeutic efficacy. Delivery of the drugs to the targeted sites at a decreased dose avoids many of the major side effects without compromising the intended therapeutic effectiveness.^127^ Lee et alused poly (ɛ-caprolactone) nanocapsule carriers to develop a sustained release nanoformulation of pilocarpine (PILO PCL NC). The release time of the drug from the capsular barrier is said to be over a period of 42 days. In comparison with pilocarpine loaded poly ɛ-caprolactone nanospheres, PILO PCL NC is observed to be almost 3 times higher in pilocarpine loading efficiency with a sustained pattern of drug release when tested *in vivo*. It showed a long-term effect in suppressing the pressure-related ocular damage in the retinal and corneal regions of the eye.^128^ Katzer et al developed prednisolone loaded nanocapsules for the treatment of ocular inflammatory disorders. The formulation has an encapsulation efficacy of 50% with the particle size ranging from 100-300 nm. The study results concluded that the nano model can regulate the release of prednisolone in a controlled manner for 5 hours without causing any significant irritation to the eye.^129^ Desai et alformulated pluronic F127 based ocular delivery system with biodegradable poly isobutyl cyanoacrylate nanocapsules for pilocarpine by interfacial polymerization technique. The formulation can deliver the drug for longer periods with increased effectiveness. The gel-like property of formulation provides sufficient adhesiveness and thereby improving the contact time with the cornea and hence bioavailability of pilocarpine.^130^

## Dendrimers

These are symmetrically shaped nanosized particles having a particle size between 1-100 nm, with reactive end group which develops into an internal cavity encapsulating the drug molecules.^131,132^ Drug delivery using dendrimers can be carried out by either enclosing a lipophilic moiety within a hydrophobic cavity or by attaching drugs onto the dendrimer surface.^133,134^ The dendrimeric structure consists of three parts: (a) Initiator core (b) Interior layers (c) Exterior functionality.^135,136^ Dendrimers are advantageous as it possesses a lower polydispersity index and the hollow core is available inside the dendrimeric structure which can be utilized for entrapping drug moieties. It also helps to target the drug delivery.^137-139^ Holden et al prepared poly amido amine dendrimer hydrogel for enhanced delivery of antiglaucoma drugs brimonidine and timolol maleate. Mucoadhesiveness and non-toxic nature of the dendrimer hydrogel makes it a suitable nanoformulation for ocular application. A sustained release of drug is observed for 56-72 hours. Overall dendrimer hydrogel formulations are found to increase the efficacy of the selected anti-glaucoma drugs.^140^ Dendrimer based targeted intravitreal therapy for attenuation of neuroinflammation and retinal degeneration using polyamidoamine drug conjugate nanodevice is developed by Iezzi et al. The drug (fluocinolone) is released for a period of 90 days from the nanodevice and is observed to be better than the free drug solution.^141^ DenTimol as a dendrimeric timolol analogue for glaucoma therapy is developed by Lancina et al. A significant decrease in the IOP (approximately 30% reduction from baseline) is observed 30 minutes after the instillation of the drug topically to Brown Norway male rats, without exhibiting any toxicity.^142^

## Hydrogel

These are highly absorbable natural or synthetic polymers formed as a three-dimensional network. The flexibility of the polymer matrix is achieved by providing sufficient water.^143^ Hydrogels swell when exposed to an aqueous environment. The three-dimensional crosslinks render these structures insoluble in water because of anionic interactions and hydrogen bonds.^136,144^ Hydrogels that releases drugs or undergo a change in their phase in response to a stimulus are widely studied.^145^ Porosity of the gel allows the timely release of the drug from the matrix structure. When used in topical ocular formulations, the hydrogels are capable to maintain muco-adhesiveness and improved ocular residence time to permit adequate action.^146^ Jung et al investigated the timolol nanoparticle loaded silicone hydrogel contact lenses for prolonged drug delivery. In the formulation, the ester bond of timolol-propoxylated glyceryl triacylate (PGT) particle is hydrolysed slowly resulting in an extended-release. The in vivo studies confirmed the extended-release of timolol with improved efficacy and safety.^147^ Kabiri et al investigated an *in situ* forming; Cannabigerolic acid (CBGA) loaded nanoparticle - laden hydrogel for ocular drug delivery in a controlled manner. CBGA is synthesized by genetic engineering methods from *E. coli*. A 300% increase in trans corneal penetration is achieved with the use of approximately 0.015% of the CBGA. The results suggested that the formulation would coat the corneal surface by blinking.^148^ Age related macular degeneration and proliferative diabetic retinopathy is treated by Avastin® (bevacizumab). Xu et alstudied the sustained release of avastin from polysaccharides cross-linked hydrogels for ocular drug delivery. To reduce the multiple application of the drug into the eye, it is modified into a hydrogel using glycol chitosan and oxidized alginate*. In vitro* studies revealed a sustained release profile with an initial burst release for 4 hours followed by a decrease in release rate.^149^ Maulvi et aldeveloped a novel implantation hydrogel contact lenses for controlled drug delivery of timolol maleate. *In vitro* release studies revealed that the implant contact lens can maintain a sustained release of drug within the therapeutic window. The implant contact lenses need to be stored at a dry state and need to be hydrated before its use to preserve the integrity of the formulation.* In vivo* experiments revealed the sustained drug release in tear fluid for a period of more than 192 h with IOP reduction without any ocular toxicity and irritancy.^150^ Hsiao et aldeveloped a depot injectable formulation to sustain the release of latanoprost for the management of glaucoma. When tested with the rabbit glaucoma model, the formulation is found to be able to lower IOP at a stable rate for a period of 40 days. The results obtained from the study prove the suitability of the system to undergo further clinical trials.^151^

## Niosomes and proniosomes

These are prepared by self-assembling the hydrated non-ionic surfactant molecules to form bilayers. The medication is encapsulated inside a vesicle surrounded by the bilayer.^152,153^ The structure of the niosomes are composed of non-ionic surfactant, cholesterol and a charge inducing molecule.^154,155^ Proniosomes are free-flowing, dry formulations of surfactant coated carriers which on hydration provides the multilamellar niosomes and provides better stability on storage, handling, transportation etc compared to niosomes.^156,157^ Proniosomes avoids the stability related problems like aggregation, fusion, and hydrolysis of the drug which is entrapped within it and improve the shelf life of the loaded moiety.^158,159^ Proniosomal gel-derived niosomes to sustain and improve the ocular delivery of brimonidine tartrate is formulated by Emad Eldeeb et al. A higher entrapment of brimonidine tartrate with sustained release profile over 24 hours is observed. The *in vivo*pharmacodynamic study of the formulation demonstrated the improved bioavailability and sustained drug release without irritation on rabbit.^160^ Research conducted by Fouda et al revealed a sustained delivery of Dorzolamide –HCl via proniosomal gel formulation. The formulation exhibited a significant reduction in IOP and increased ocular availability of dorzolamide in comparison with the marketed eye drops.^161^ Proniosomes are employed for the delivery of Lomefloxacin hydrochloride for the treatment of bacterial conjunctivitis. The proniosomal gel preparations of the drug increased the therapeutic efficacy, the penetration properties and retention time of the loaded moiety. The entrapment efficiency of the formulation is found to be greater than 80%, a controlled drug release rate for 12 hours is observed and is found to be stable for three months when tested *in vitro*.^162^

## Conclusion

Glaucoma is a chronic ailment that poses a great threat to vision, if not identified and treated immediately. Early diagnosis of the disease and maintaining strict medication adherence is necessary to prevent the advancement of the disease. In this review, a brief outline is given regarding glaucoma, with its pathology and treatment methods available. The review focuses on newer delivery approaches available for glaucoma with their advantages. The current treatment strategy for glaucoma is based on the sole reduction in the IOP by topically applying agents like prostaglandins, alpha agonists, carbonic anhydrase inhibitors etc. Certain new and emerging technologies involving the use of nanomedicine are being developed which may prove beneficial to the diseased society.

## Ethical Issues

Not applicable

## Conflict of Interest

The authors have no affiliation or ﬁnancial conflict with the subject of materials discussed in the manuscript with any of the organization or entity.
